# The role of exosomes in the pathogenesis and management of diabetic kidney disease: a systematic review and meta-analysis

**DOI:** 10.3389/fendo.2024.1398382

**Published:** 2024-12-03

**Authors:** Yan Zheng, Chu Xu, Yan Jin

**Affiliations:** Department of Endocrinology, Zhoushan Hospital, Zhejiang Province, Zhoushan, Zhejiang, China

**Keywords:** exosomes, diabetic kidney disease, pathogenesis, management, meta-analysis

## Abstract

**Objective:**

This systematic review and meta-analysis aimed to synthesize the role of exosomes in the pathogenesis and management of diabetic kidney disease.

**Methods:**

PubMed, Embase, Cochrane Library, and Web of Science were searched for studies that compared the levels of exosomes between patients with diabetic kidney disease and controls published up to 27 November 2023. Methodological quality was assessed using the JBI Appraisal Checklist for Case–Control Studies. The methodology of the samples and the main results were summarized. A meta-analysis of the diagnostic performance of exosomes was performed using estimates of test sensitivity and specificity, and these values were summarized using summary receiver-operating characteristic curves. The results were reported following the PRISMA 2020 checklist.

**Results:**

A total of 32 studies, including 1,119 patients with diabetic kidney disease and 1,328 controls, met the inclusion criteria. A total of 78 upregulated and 22 downregulated microRNAs, 2 upregulated and 4 downregulated mRNAs, 6 upregulated and 1 downregulated proteins, and 4 upregulated lipids were identified. The miR-126, miR-145, miR-150, miR-21, and WT1 mRNA dysregulation were consistently reported in at least two studies. The overall sensitivity and specificity of the exosomes in diabetic kidney disease diagnosis were 0.70 (95% CI: 0.59–0.80) and 0.79 (95% CI: 0.70–0.85), respectively. The summary receiver operating characteristic curve was plotted to assess diagnostic accuracy with the area under the curve (AUC) of 0.82 (95% CI: 0.78–0.85).

**Conclusion:**

Exosomes have great potential to become effective diagnostic biomarkers for diabetic kidney disease. Panels of exosomes or the combination of exosomes with other clinical indicators seemed more accurate than single exosomes.

## Introduction

Diabetic kidney disease (DKD) is a frequent microvascular complication of type 1 and type 2 diabetics. Approximately 40% of diabetic patients eventually develop DKD, which has been associated with an increased incidence of pain, falls, and reduced quality of life (1). It is also the most common cause of end-stage renal disease requiring renal replacement therapy, which is associated with high mortality and morbidity (2–4). The mortality risk in patients with DKD is 31.1% higher compared to diabetic patients. The mortality risk is even higher in incipient DKD patients, imposing substantial public health and economic burdens (5, 6). DKD is often undiagnosed until the manifestations of serious complications, inhibiting timely medical management to control disease progression (4, 7). Persistently elevated albumin excretion [albumin-to-creatine ratio (ACR) ≥ 30 mg/g] and low estimated glomerular filtration rate (eGFR < 60 ml/min/1.73 m^2^) are standard diagnostic indicators for DKD in a clinical setting. Still, these indicators have limited specificity and predictive power (8, 9). Kidney biopsy is superior in differentiating DKD from non-DKD and provides better risk stratification of DKD than the routine measurement of ACR and eGFR (1, 10). However, due to its invasiveness and patient burden, kidney biopsy is not feasible for the routine practice of DKD management.

Exosomes are membranous extracellular vesicles with a nanostructure and diameters ranging from 30 to 150 nm (11). Studies have found that exosomes act as messengers in cell–cell communication by transferring content to the target cells’ cytoplasm and altering the recipient cells’ physiological state (12). The generation of exosomes begins from endocytosis to form early endosomes by inward budding of the plasma membrane triggered by external stimuli or microbial attacks. After that, exosomes are shed into various body fluids and widely distributed in almost all kinds of body fluids, suggesting an irreplaceable role of exosomes in physiological and pathological conditions (13). During exosome biogenesis and release, selective cargo loading occurs, and particular cellular constituents are shuttled into exosomes containing various microRNAs (miRNAs), mRNAs, DNAs, lipids, and many other cellular components (14). Exosomes transfer autocrine or paracrine signals by a cell–cell crosstalk between kidney resident cells. High concentrations of glucose and the stimulated renal cells can lead to changes in composition and communication, further changing and damaging intact cells, which suggests that exosomes packaged with functional cargo have a vital role in diverse cellular processes and diseases, including DKD (15). Exosomes can be isolated from body fluid, including blood, urine, and saliva, making them ideal candidates for the non-invasive diagnosis of DKD (16, 17).

A thorough literature search identified three published review studies investigating the exosome biomarkers in DKD, particularly miRNA (9, 18, 19). Several dysregulated miRNAs were identified in DKD, and it was shown that specific miRNAs were significantly associated with clinical indicators of DKD, including HbAc1, ACR, and eGFR, suggesting important diagnostic and pathogenetic implications. However, the existing reviews failed to include standard components in evaluating exosomes other than functional miRNA (i.e., mRNA, long non-coding RNA, proteins) (16) or to investigate the diagnostic value of exosomes using meta-analysis.t Considering that it is challenging to inform clinical decisions without evidence related to the accuracy and sensitivity of the diagnostic tests, we conducted a systematic review and meta-analysis to synthesize evidence on clinical outcomes of all exosome types in DKD. We also analyzed the role of exosomes as biomarkers of DKD. Our main aim was to further elucidate the role of exosomes in the pathogenesis and management of DKD.

## Methods

### Search strategy

Databases, including PubMed, Embase, Cochrane Library, and Web of Science, were searched to identify eligible studies on exosomes in DKD published from the inception of the database until 27 November 2023. The search strategy was developed using the key terms “exosome” and “extracellular vesicle” in combination with “diabetic kidney disease” and “diabetic nephropathy.” The detailed search strategy for each database is listed in **Supplementary Appendix 1**. Two reviewers independently screened the titles and abstracts against the inclusion criteria. After identifying potentially relevant records, the two reviewers screened all full-text records. The inclusion criteria were as follows: 1) patients diagnosed with DKD; 2) studies that evaluated exosomes using blood, urine, or other samples and compared the levels between diabetic kidney disease patients and controls (i.e., diabetic patients without nephropathy or healthy individuals); 3) cohort studies, case–control studies, and interventional studies; and 4) studies published in the English language. The exclusion criteria were 1) duplicated studies; 2) animal studies or *in-vitro* experiments; 3) reviews, conference proceedings, comments, or case reports; and 4) data of interest cannot be extracted, or full text is unavailable.

### Data extraction and quality assessment

The following data were extracted for each included article: author, year of publication, study design, country, number of samples (case vs. control), source of sample (i.e., blood, urine), method of extraction, cutoff criteria, exosome information, study outcomes (up- or downregulation), and potential diagnostic marker of DKD. If the study conducted the diagnostic test, sensitivity, specificity, or true positive (TP), false positive (FP), false negative (FN), and true negative (TN) were extracted. The two reviewers appraised the quality and risk of bias of the included studies according to the Joanna Briggs Institute (JBI) Appraisal Checklist for Case–Control Studies (20). The checklist includes 10 items: evaluating the appropriateness of the cases and controls, exposure measurement, confounding factors, outcome assessment, and statistical analysis methods. Criteria were classified as “yes,” “no,” “unclear,” or “not applicable (NA).” In the case of conflicting evaluations, the agreement was reached after discussion.

### Data synthesis and statistical analysis

The results of the included studies were synthesized by the direction of dysregulation and the type of exosome. Meta-analysis of diagnostic tests was performed using STATA v.17 (College Station, TX, USA) with the MIDAS module. The estimated pooled sensitivity and specificity of exosomes in DKD diagnosis with a 95% confidence interval (CI) were calculated using extracted TP, FP, FN, and TN in each included study, and bivariate random-effects models and forest plots for sensitivity and specificity were generated. The summary receiver operating characteristic (SROC) and the area under the curve (AUC) were plotted and calculated, assessing exosome pooled diagnostic value. Heterogeneity between studies was evaluated using the chi-square test, and *I* ^2^ >50% represented a high degree of heterogeneity. Due to the limited number of studies (*n* < 10) included in the meta-analysis, the publication bias was not assessed as recommended by the Cochrane Handbook (21).

## Results

**Figure 1:**
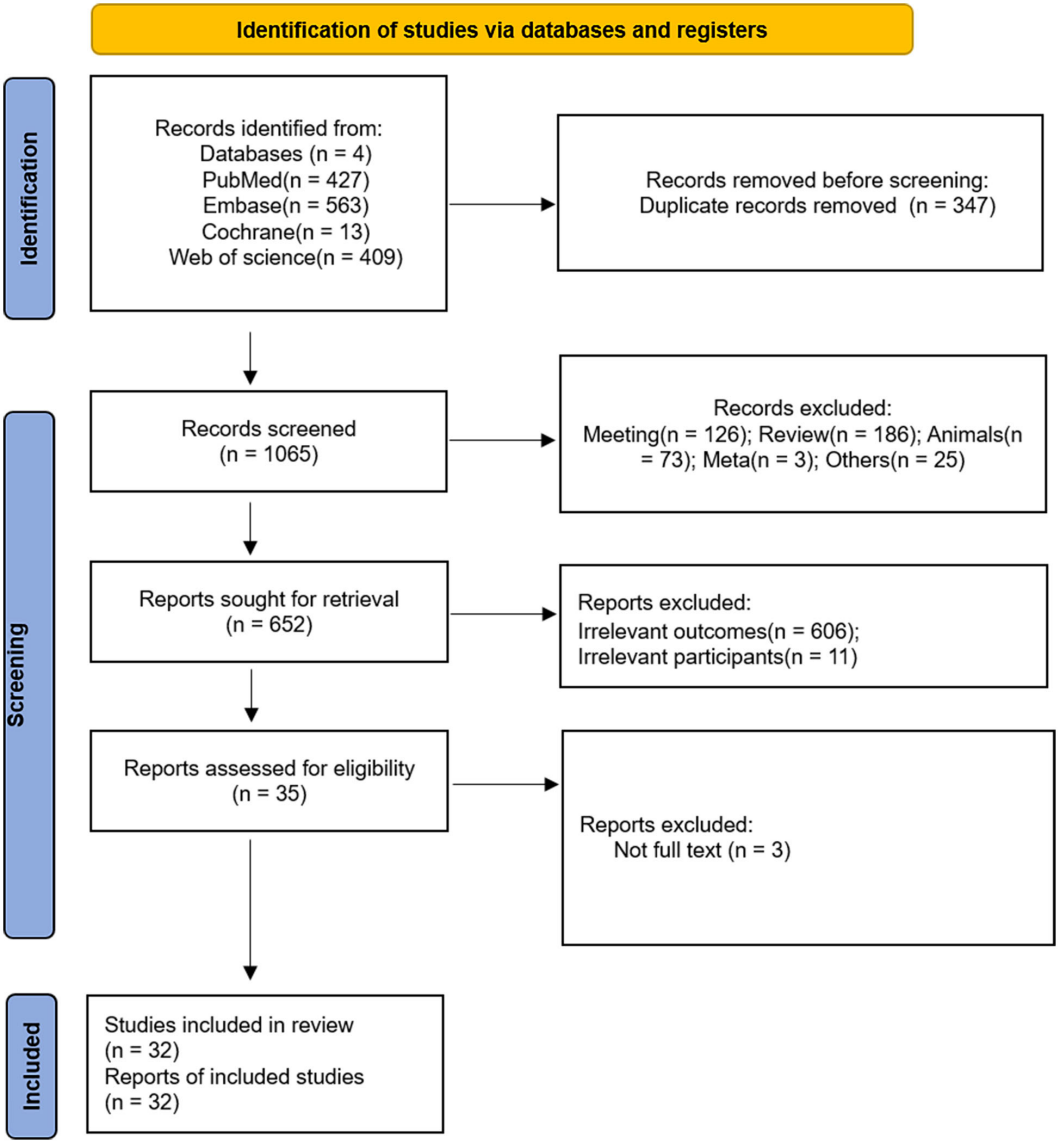
PRISMA flowchart.

After duplicates were removed, 1,065 records were identified. Among these, 413 were excluded (conference proceedings, reviews, animal studies, etc.), and the remaining 652 studies were further screened against the inclusion and exclusion criteria. After excluding 606 studies with irrelevant outcomes, 11 with outside participants, and 3 with no full-text available, 32 eligible publications with 1,119 patients with DKD and 1,328 without DKD were included in the systematic review (16, 22–52). The PRISMA flowchart is presented in **Figure 1**. The characteristics of the included studies are summarized in **Table 1**. Most included studies (30/32; 93.8%) were case–control studies assessing the levels of exosomes in DKD and controls. In their study, Almquist et al. investigated the effects of simvastatin alone or with ezetimibe on microparticles in patients with or without DKD using a randomized controlled design (31). Sun et al. described a two-stage randomized controlled study, and in the second stage, they evaluated the potential role of urine exosomes as early diagnostic biomarkers for DKD (16). The majority of the included studies were conducted in China (15/32; 46.9%), followed by India (3/32; 9.4%), Poland (2/32; 6.3%), the Netherlands (2/32; 6.3%), and Italy (2/32; 6.3%). Twenty-three studies (71.9%) recruited patients with type II DKD only, three studies (9.4%) recruited patients with type I DKD only, and one study (3.1%) included both types I and II. In comparison, five studies (15.6%) did not specify the etiology of included DKD patients. With regard to the source of the sample, 23 studies (71.9%) used urine samples, 9 (28.1%) involved plasma or serum, and 1 study (3.1%) used lipids. qRT-PCR was the most frequently used method for detecting and measuring exosomes (18/32; 56.3%). Most included studies used *P <*0.05 as the cutoff value (20/32; 62.5%), while others used fold change values as the cutoff criteria (10/32; 31.3%).

**Table 1:** Characteristics of the included studies.

Author	Year of publication	Country	Disease	No. of samples (DN/control)	Sample	Methods	Cutoff criteria
Abe et al. (22)	2018	Japan	T2DN	25 (20/5)	Urine	qRT-PCR	*P* < 0.001
Almquist et al. (31)	2016	Sweden	T1DN and T2DN	39 (18/21)	Plasma	Flow cytometry	*P* < 0.05
Barutta et al. (32)	2013	Italy	T1DN	34 (12/22)	Urine	qRT-PCR	*P* < 0.05
Cai et al. (23)	2020	China	DN^a^	78 (17/61)	Urine	Flow cytometry	*P* < 0.05
Dehghanbanadaki et al. (24)	2022	Iran	T2DN	196 (88/108)	Urine and plasma	qRT-PCR	*P* < 0.05
Delić et al. (25)	2016	Germany	T2DN	24 (8/16)	Urine	qRT-PCR	FC > 2; *P* < 0.05
Dimuccio et al. (33)	2022	Italy	T2DN	46 (29/17)	Urine	qRT-PCR	*P* < 0.05
Feng et al. (34)	2021	China	T2DN	71 (32/39)	Urine	qRT-PCR	*P* < 0.05
Florijn et al. (26)	2019	Netherlands	T1DN	45 (19/26)	Plasma	qRT-PCR, ELISA, and Western blot	*P* < 0.05
Gu et al. (35)	2023	China	DN^a^	75 (30/45)	Urine	Bradford assay	*P* < 0.05
Hashemi et al. (27)	2021	Iran	T2DN	256 (103/153)	Plasma	qRT-PCR	*P* < 0.05
Jia et al. (28)	2016	China	T2DN	90 (50/40)	Urine	qRT-PCR and Western blot	*P* < 0.05
Kalani et al. (29)	2013	Italy	T1DN	73 (18/55)	Urine	Western blot	*P* < 0.05
Kamińska et al. (30)	2016	Poland	T2DN	70 (15/55)	Urine	TRF assay	*P* < 0.05
Kim et al. (36)	2019	South Korea	DN^a^	74 (23/51)	Serum	qRT-PCR	FC > 2; *P* < 0.05
Kumari and Singh (37)	2018	India	DN^a^	20 (10/10)	Lipid	LC-MS	FC > 1.5; *P* < 0.05
Li et al. (1) (38)	2023	China	T2DN	132 (44/88)	Urine	Western blot and ELISA	FC > 1.5; *P* < 0.05
Li et al. (2) (39)	2023	China	T2DN	144 (48/96)	Urine	MS, Western blot, and ELISA	FC > 1.5; *P* < 0.05
Lou et al. (40)	2017	China	T2DN	131 (54/77)	Urine	ELISA	*P* < 0.05
Pan et al. (41)	2022	China	T2DN	80 (40/40)	Plasma	Western blot and LC-ESI-MS	NR
Prabu et al. (42)	2019	India	T2DN	160 (80/80)	Urine	qRT-PCR	*P* < 0.05
Rodrigues et al. (43)	2018	Brazil	T2DN	69 (39/30)	Plasma	Flow cytometry	*P* < 0.05
Sinha et al. (44)	2023	India	T2DN	17 (9/8)	Urine	Flow cytometry, Western blot, and qRT-PCR	*P* < 0.05
Sun et al. (45)	2012	China	T2DN	161 (84/77)	Urine and serum	ELISA	*P* < 0.05
Sun et al. (16)	2017	China	DN^a^	62 (62/0)	Urine	Flow cytometric	*P* < 0.05
Uil et al. (46)	2021	Netherlands	T2DN	92 (61/31)	Plasma	Flow cytometry and qRT-PCR	*P* < 0.05
Wang et al. (47)	2023	China	T2DN	63 (42/21)	Urine	qRT-PCR	*P* < 0.05
Xie et al. (48)	2017	China	T2DN	10 (5/5)	Urine	qRT-PCR	FC > 2; *P* < 0.05
Zang et al. (49)	2019	China	T2DN	66 (36/30)	Urine	qRT-PCR	FC > 1.5; *P* < 0.05
Zapała et al. (50)	2023	Poland	T2DN	14 (8/6)	Urine	qRT-PCR	FC > 2
Zhao et al. (51)	2020	China	T2DN	6 (3/3)	Urine	qRT-PCR	FC > 2; *P* < 0.05
Zhao et al. (52)	2023	China	T2DN	24 (12/12)	Urine	NGS	FC > 5

DN, diabetic neuropathy; TRF, time-resolved fluorometry; LC-MS, liquid chromatography-mass spectrometry; LC-ESI-MS, liquid chromatography-electrospray ionization-mass spectrometry; NGS, next-generation sequencing; FC, fold change; NR, not reported.

a Studies did not specify type 1 or type 2 diabetes.

### Quality assessment

The quality assessment ratings of the included studies are listed in **Table 2**. A total of 11 studies were rated “no” for item 1 (Were the groups comparable other than the presence of disease in cases or the absence of disease in controls)?, mainly due to the significant age and blood pressure difference between groups. Two studies were rated “unclear” for item 1 because they failed to report patients’ demographic and clinical characteristics. For item 2 (Were cases and controls matched appropriately)?, five studies were rated “unclear” as they did not include a clear definition of the source population. All studies were positively evaluated for items 3, 4, and 5, referring to the identification of cases/controls and measure exposure. Only five studies received favorable ratings for items 6 and 7 for developing strategies to deal with confounding factors (e.g., multivariate regression analysis). All studies were positively rated for items 8, 9, and 10 regarding outcome measurement and statistical analysis.

**Table 2:** Quality assessment.

Study	1. Were the groups comparable other than the presence of disease in cases or the absence of disease in controls?	2. Were cases and controls matched appropriately?	3. Were the same criteria used for the identification of cases and controls?	4. Was exposure measured in a standard, valid, and reliable way?	5. Was exposure measured in the same way for cases and controls?	6. Were confounding factors identified?	7. Were strategies to deal with confounding factors stated?	8. Were outcomes assessed in a standard, valid, and reliable way for cases and controls?	9. Was the exposure period of interest long enough to be meaningful?	10. Was appropriate statistical analysis used?
Abe2018	N	Y	Y	Y	Y	N	NA	Y	Y	Y
Almquist 2016	Y	Y	Y	Y	Y	Y	Y	Y	Y	Y
Barutta2013	Y	Y	Y	Y	Y	N	NA	Y	Y	Y
Cai2020	N	Y	Y	Y	Y	Y	Y	Y	Y	Y
Dehghanbanadaki2022	Y	Y	Y	Y	Y	N	NA	Y	Y	Y
Delić2016	Y	Unclear	Y	Y	Y	Y	N	Y	Y	Y
Dimuccio2022	N	Y	Y	Y	Y	N	NA	Y	Y	Y
Feng2021	N	Y	Y	Y	Y	N	NA	Y	Y	Y
Florijn2019	Unclear	Unclear	Y	Y	Y	Y	Y	Y	Y	Y
Gu2023	Y	Y	Y	Y	Y	Y	Y	Y	Y	Y
Hashemi2021	N	Y	Y	Y	Y	N	NA	Y	Y	Y
Jia2016	Y	Y	Y	Y	Y	N	NA	Y	Y	Y
Kalani2013	Y	Unclear	Y	Y	Y	N	NA	Y	Y	Y
Kamińska2016	Y	Unclear	Y	Y	Y	N	NA	Y	Y	Y
Kim2019	N	Y	Y	Y	Y	N	NA	Y	Y	Y
Kumari2018	Y	Y	Y	Y	Y	N	NA	Y	Y	Y
Li2023 (1)	Y	Y	Y	Y	Y	Y	Y	Y	Y	Y
Li2023 (2)	Y	Y	Y	Y	Y	N	NA	Y	Y	Y
Lou2017	Y	Y	Y	Y	Y	N	NA	Y	Y	Y
Pan2022	N	Unclear	Y	Y	Y	N	NA	Y	Y	Y
Prabu2019	N	Y	Y	Y	Y	N	NA	Y	Y	Y
Rodrigues2018	N	Y	Y	Y	Y	N	NA	Y	Y	Y
Sinha2023	Unclear	Y	Y	Y	Y	N	NA	Y	Y	Y
Sun2012	Y	Y	Y	Y	Y	N	NA	Y	Y	Y
Sun2016	Y	Y	Y	Y	Y	N	NA	Y	Y	Y
Uil2021	Y	Y	Y	Y	Y	N	NA	Y	Y	Y
Wang2023	N	Y	Y	Y	Y	N	NA	Y	Y	Y
Xie2017	Y	Y	Y	Y	Y	N	NA	Y	Y	Y
Zang2019	N	Y	Y	Y	Y	N	NA	Y	Y	Y
Zapała2023	Y	Y	Y	Y	Y	N	NA	Y	Y	Y
Zhao2020	Y	Y	Y	Y	Y	N	NA	Y	Y	Y
Zhao2023	Y	Y	Y	Y	Y	N	NA	Y	Y	Y

### Exosomes of diabetic kidney disease

The results of the included studies on exosomes are summarized in **Table 3**. A total of 78 upregulated and 22 downregulated miRNAs in DKD patients were identified in 14 studies. Four dysregulated miRNAs were reported in at least two different studies: miR-126 (26, 33, 50), miR-145 (32, 33), miR-150 (36, 48), and miR-21 (49, 50, 52). Two upregulated mRNAs (WT1 and CCL21) and four downregulated mRNAs (CDH2, MCP-1, PAI-1, and ACE) were identified in four studies; the upregulation of WT1 was reported in two studies (22, 27). Six upregulated proteins (WT1, CALM1, PAK6, EGFR, SHC1, and uromodulin) and one downregulated protein (CD63) were identified in five studies. Almquist et al. and Rodrigues et al. reported that the total levels of microparticles and subgroups were higher in DKD patients than in controls (31, 43). Furthermore, Cai et al. found that DKD patients had greater numbers of urinary microvesicles (MVs) from podocytes, proximal tubular cells, and endothelial cells than controls. Gu et al. found that the protein concentration of urinary extracellular vesicles (EVs) increased in DKD (35). Kamińska et al. reported that the density of EVs decreased in DKD (30), and Pan et al. identified the up- and downregulation of EVs in DKD (41). Kumari and Singh found the upregulation of DG, TG, GM3, and LysoPC lipids in DKD patients.

**Table 3:** The results of exosomes and potential biomarkers of diabetic neuropathy in the included studies.

Study	Results	Potential diagnostic markers of DN
Abe et al., 2018 (22)	WT1 mRNA ↑ in DN compared with MCNS and controls	WT1 mRNA^a^ ♢AUC: 0.705
Almquist et al., 2016 (31)	Total levels of MPs and subpopulations of MPs: PMPs, MMPs, and EMPs ↑ in DN compared with DM	NR
Barutta et al., 2013 (32)	miR-130a and miR-145 ↑ in DN compared with DM and controls; miR-155 and miR-424 ↓ in DN compared with DM and controls	NR
Cai et al., 2020 (23)	MVs from podocytes, proximal tubular cells, and endothelial cells ↑ in DN compared with controls	Podocyte nephrin+ MVs and diabetic retinopathy ♢AUC: 0.899 (95% CI: 0.821–0.977), sensitivity: 88.9%, specificity: 89.7%
Dehghanbanadaki et al., 2022 (24)	CDH2 and MCP-1 mRNA ↓ in overt DN and incipient DN compared to DM; PAI-1 mRNA ↓ in incipient DN compared to controls	1/CDH2 mRNA ♢AUC: 0.61 (95% CI: 0.50–0.71), sensitivity: 37.7%, specificity: 83.9% 1/MCP-1 mRNA ♢AUC: 0.61 (95% CI: 0.51–0.71), sensitivity: 69.8%, specificity: 61.3% 1/CDH2 mRNA^a^ ♢AUC: 0.75 (95% CI: 0.65–0.85), sensitivity: 74.3%, specificity: 69.4%, 1/MCP-1 mRNA^a^ ♢AUC: 0.66 (95% CI: 0.55–0.77), sensitivity: 57.1%, specificity: 74.2%
Delić et al., 2016 (25)	miR-320c, miR-6068, miR-1234-5p, miR-6133, miR-4270, miR-4739, miR-371b-5p, miR-638, miR-572, miR-1227-5p, miR-6126, miR-1915-5p, miR-4778-5p, and miR-2861 ↑ in DN compared to DM and controls; miR-30d-5p and miR-30e-5p ↓ in DN compared to DM and controls	NR
Dimuccio et al., 2022 (33)	miR145 and miR126 ↑ in DN compared to DM	NR
Feng et al., 2021 (34)	CCL21 mRNA ↑ in DN compared to DM	CCL21 mRNA ♢AUC: 0.888 (95% CI: 0.752–1) CCL21 mRNA^a^ ♢AUC: 1.0 (95% CI: 1.0–1.0), sensitivity: 100%, specificity: 100%
Florijn et al., 2019 (26)	miR-21, miR-126, and miR-660 ↑ in DN compared to controls; miR-132 ↓ in DN compared to controls	NR
Gu et al., 2023 (35)	The protein concentration of uEVs in DN ↑ compared to controls	NR
Hashemi et al., 2021 (27)	WT1 mRNA ↑ in DN compared with DM and controls; ACE mRNA ↓ in DN compared with DM and controls	WT1 mRNA ♢AUC: 0.63 (95% CI: 0.55–0.72), sensitivity: 50%, specificity: 74% 1/ACE mRNA ♢AUC: 0.62 (95% CI: 0.54–0.71), sensitivity: 65.2%, specificity: 61% WT1 mRNA^a^ ♢AUC: 0.83 (95% CI: 0.74–0.92), sensitivity: 67.6%, specificity: 93% 1/ACE mRNA^a^ ♢AUC: 0.75 (95% CI: 0.66–0.83), sensitivity: 73%, specificity: 72%
Jia et al., 2016 (28)	miR-192, miR-194, and miR-215 ↑ in incipient DN compared to DM and controls	miR-192 ♢AUC: 0.802 (95% CI: 0.696–0.907) miR-194 ♢AUC: 0.703 (95% CI: 0.581–0.826) miR-215 ♢AUC: 0.757 (95% CI: 0.545–0.869)
Kalani et al., 2013 (29)	WT1 protein ↑ in DN compared to DM	WT1 protein ♢AUC: 0.92 (95% CI: 0.83–0.99), sensitivity: 88.6%, specificity: 100%
Kamińska et al., 2016 (30)	Density of EVs ↓ in DN compared to DM	NR
Kim et al., 2019 (36)	miR-4449, miR-1246, miR-642a-3p, let-7c-5p, miR-1255b-5p, let-7i-3p, miR-5010-5p, and miR-150-3p ↑ in DN compared to controls	NR
Kumari and Singh, 2018 (37)	DG, TG, GM3, and LysoPC lipids ↑ in DN compared to DM	NR
Li et al., 2023 (38) (1)	CALM1 protein ↑ in DN compared to DM and controls	CALM1 ♢AUC: 0.903 (95% CI: 0.826–0.979) CALM1 and serum ALB ♢AUC: 0.931 (95% CI: 0.863–1.000)
Li et al., 2023 (39) (2)	PAK6, EGFR, and SHC1 protein ↑ in DN compared to DM	PAK6 ♢AUC: 0.829 (95% CI: 0.728–0.929) EGFR ♢AUC: 0.797 (95% CI: 0.683–0.912) PAK6 and EGFR ♢AUC: 0.897 (95% CI: 0.824–0.970)
Lou et al., 2017 (40)	Microvesicle-bound uromodulin (protein) ↑ in DN compared to DM and controls	NR
Pan et al., 2022 (41)	Uracil, 4-acetamidobutyric acid, and ectoine (EVs) ↑ in DN compared to DM; pyrazine, PE (20:4(5Z,8Z,11Z,14Z)/P-18:1(11Z)), Cer (d18:1/24:1(15Z)), EPA, sphingosine 1-phosphate, PC (O-16:0/0:0), and LPC (O-18:1/0:0) ↓ in DN compared to DM	Uracil, LPC (O-18:1/0:0), S1P, and 4-acetamidobutyric acid ♢AUC: 0.944
Prabu et al., 2019 (42)	miR-27b-3p and miR-135b-5p ↑ in DN compared to DM	let-7i-5p, miR-15b-5p, miR-24-3p, and miR-27b-3p ♢AUC: 0.867 let-7i-5p, miR-15b-5p, miR-24-3p, and miR-27b-3p^a^ ♢AUC: 0.986
Rodrigues et al., 2018 (43)	PMPs, LMPs, EMPs, and TFMPs ↑ in DN compared to controls	NR
Sinha et al., 2023 (44)	miR-155-5p, miR-28-3p, and miR-425-5p ↑ in DN compared to controls; miR-663a ↓ in DN compared to controls	NR
Sun et al., 2012 (45)	Urinary MV-DPP IV ↑ in DN compared to controls	NR
Sun et al., 2017 (16)	CD63 (tetraspanin; protein) ↓ in DN compared to DM	NR
Uil et al., 2021 (46)	miR-99a-5p, miR-205-5p, and miR-124-3p↑ in DN compared to DM; miR-136-5p, miR-744-5p, miR-625-3p, and miR-19b-3p ↓ in DN compared to DM	NR
Wang et al., 2023 (47)	miR-615-3p ↑ in DN compared to DM and controls	miR-615-3p ♢AUC: 0.743 (95% CI: 0.638–0.849) miR-3147 ♢AUC: 0.582 (95% CI: 0.459–0.705) miR-615-3p and urine albumin-to-creatinine ratio ♢AUC: 0.974 (95% CI: 0.934–1.000)
Xie et al., 2017 (48)	miR-362-3p, miR-877-3p, and miR-150-5p ↑ in DN compared to DM; miR-15a-5p ↓ in DN compared to DM	NR
Zang et al., 2019 (49)	miR-21-5p, let-7e-5p, and miR-23b-3p ↑ in DN compared to DM; miR-30b-5p and miR-125b-5p ↓ in DN compared to DM	miR-30b-5p, miR-21–5p, age, gender, HDL-C ♢AUC: 0.932 (95% CI: 0.853–1.000)
Zapała et al., 2023 (50)	miR-514a-5p, miR-451a, miR-548z, miR-548h-3p, miR-214-3p, miR-514b-5p, miR-148b-5p, miR-1269a, miR-4802-3p, miR-126-3p, miR-378f, miR-342-5p, miR-450a-5p, miR-1307-3p, miR-503, and miR-542-5p ↑ in DN compared to controls; miR-21-3p, miR-4792, miR-375, miR-1268a, miR-501-5p, miR-19b-1-5p, miR-378a-5p, miR-582-5p, and miR-545-3p ↓ in DN compared to controls	NR
Zhao et al., 2020 (51)	miR-4491, miR-2117, miR-4507, miR-5088-5P, miR-1587, miR-219a-3p, miR-5091, miR-498, miR-4687-3p, miR-516b-5p, miR-4534, miR-1275, miR-5007-3p, and miR-4516 ↑ in DN compared to DM and controls	miR-4534 ♢AUC: 0.786 (95% CI: 0.607–0.965), sensitivity: 85.7%, specificity: 78.6%
Zhao et al., 2023 (52)	miR-21-5p, miR-378a-3p, miR-486-5p, and miR-22-3p ↑ in DN compared to DM; miR-215-5p ↓ in DN compared to DM	NR

WT1, Wilms tumor 1; MCNS, minimal change nephrotic syndrome; PMPs, MMPs, and EMPs, platelet, monocyte, and endothelial microparticles; MVs, microvesicles; uEVs, urinary extracellular vesicles; ALB, albumin; CALM1, calmodulin-1; PAK6, serine/threonine-protein kinase PAK6; EGFR, epidermal growth factor receptor; SHC1, SHC-transforming protein 1; TFMPs, expressing tissue factor; DPP-IV, microvesicle-dipeptidyl peptidase-IV; HDL-C, high-density lipoprotein cholesterol; NR, not reported.

a Overt DN detection.

↓ means the down-regualtion and ↑ means the up-regulation.

### The role of exosomes as biomarkers of diabetic kidney disease

Fourteen studies conducted 19 diagnostic tests of exosomes on DKD; the outcomes are summarized in **Table 3**. Five studies investigated the role of miRNA as a diagnostic biomarker for DKD (28, 42, 47, 49, 51), of which six diagnostic tests investigated the diagnostic value of single miRNAs (miR-192, miR-194, miR-215, miR-615-3p, miR-3147, miR-4534), and the AUCs ranged from 0.582 to 0.802 (28, 47, 51). Prabu et al. combined the EV levels of let-7i-5p, miR-15b-5p, miR-24-3p, and miR-27b-3p to discriminate non-DKD diabetic patients from DKD patients (AUC: 0.867) and non-DKD diabetic patients from overt DKD patients (AUC: 0.986) (42). Two studies investigated the diagnostic value of miRNA in combination with other clinical indicators [miR-615-3p and ACR, AUC: 0.974; miR-30b-5p, miR-21-5p, age, gender, and high-density lipoprotein cholesterol (HDL-C), AUC: 0.932] (47, 49). Four studies investigated the role of mRNA as a diagnostic biomarker for DKD (22, 24, 27, 34); Abe et al. and Hashemi et al. both assessed WT1 as a biomarker for DKD (AUC: 0.63–0.705) (22, 27), and Hashemi et al. also evaluated WT1 as a biomarker for overt DKD (AUC: 0.83) (27). The other studied mRNAs included CDH2 (AUC: 0.61 for DKD and 0.75 for overt DKD), MCP-1 (AUC: 0.61 for DKD and 0.66 for overt DKD), CCL21 (AUC: 0.888 for DKD and 1.0 for overt DKD), and ACE (AUC: 0.62 for DKD and 0.75 for overt DKD) (24, 27, 34). Three studies investigated the diagnostic value of proteins for DKD: WT1 (AUC: 0.92), CALM1 (AUC: 0.903), CALM1 and serum albumin (AUC: 0.931), PAK6 (AUC: 0.829), EGFR (AUC: 0.797), and PAK6 and EGFR (AUC: 0.897) (29, 38, 39). Two remaining studies assessed podocyte nephrin+ MVs and diabetic retinopathy (AUC: 0.899) and the combination of uracil, LPC (O-18:1/0:0), S1P, and 4-acetamido butyric acid (AUC: 0.944) as diagnostic biomarkers of DKD (23, 41).

**Figure 2:**
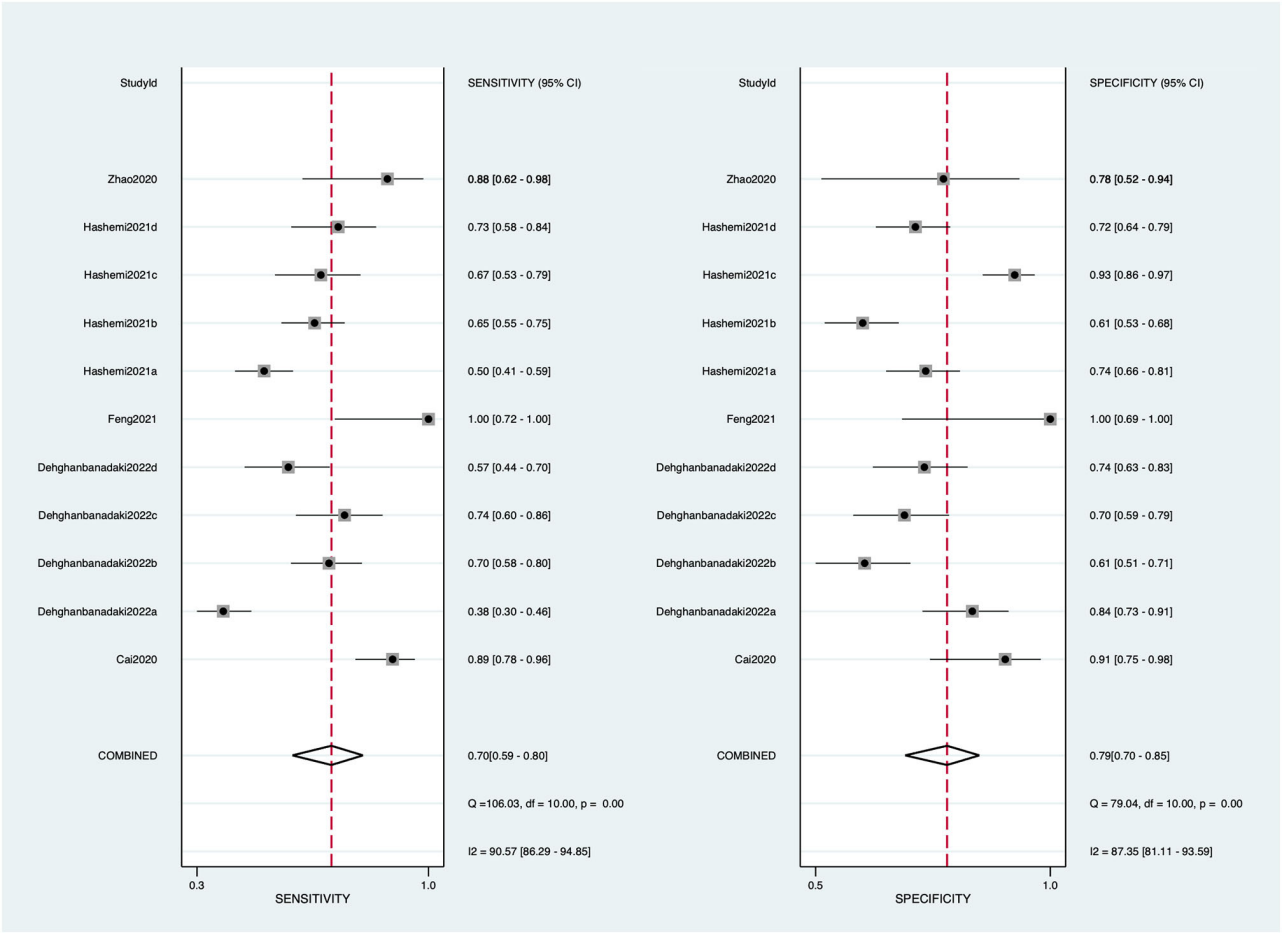
Forest plots of sensitivity and specificity on exosomes used to diagnose diabetic kidney disease. Square symbols represent the sensitivity or specificity of each study according to the Study ID shown on the *y*-axis, while the short lines cutting through represent the relative 95% CI. The diamond symbols refer to the combined sensitivity or specificity. A “COMBINED” label coordinating to the diamond symbol is shown on the *y*-axis underneath all Study IDs.

**Figure 3:**
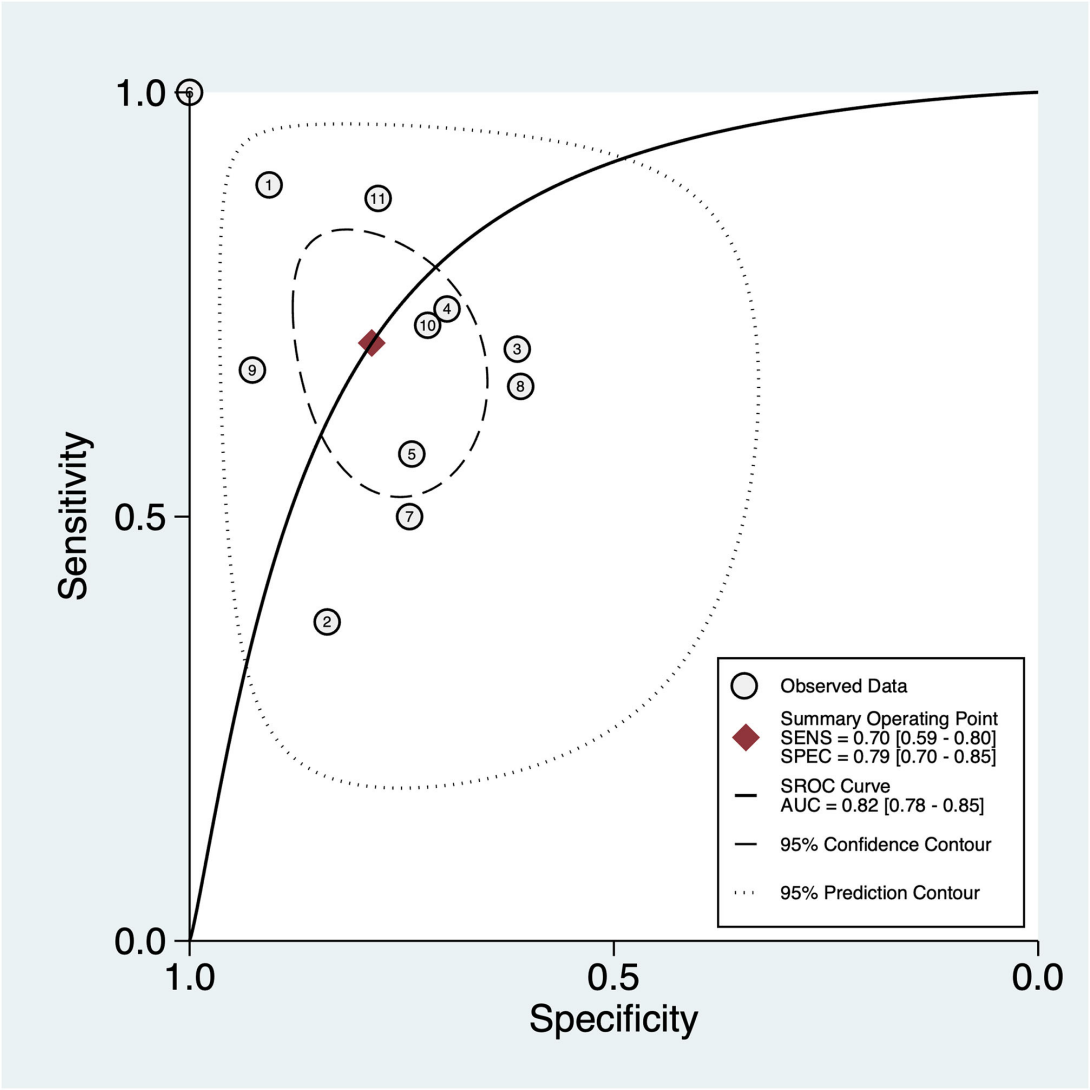
The SROC of sensitivity and specificity of exosomes for the prediction of DKD with the data of 11 reports from 5 studies.

Eleven diagnostic tests of five studies reported the sensitivity, specificity, and AUC clearly and were subsequently included in the meta-analysis (23, 24, 27, 34, 51). The pooled sensitivity and specificity with their 95% CIs (**Figure 2**) and the AUC (**Figure 3**) were 0.70 (95% CI: 0.59–0.80), 0.79 (95% CI: 0.70–0.85), and 0.82 (95% CI: 0.78–0.85), indicating that exosomes had good accuracy and efficiency in diagnosing DKD and suggesting that they are a promising alternative to the traditional diagnostic method, such as ACR and eGFR.

## Discussion

This systematic review and meta-analysis identified 110 unique exosomes (i.e., miRNAs, mRNAs, proteins, and lipids) that were significantly dysregulated in DKD patients. In the meta-analysis of DKD diagnostic tests, exosomes had good sensitivity (0.70) and specificity (0.78). The AUC was 0.82 for the SROC curve, indicating excellent overall diagnostic accuracy.

Approximately half of the included studies investigated the role of miRNA in DKD management, and a total of 78 upregulated and 22 downregulated miRNAs were identified in our review. Four miRNA families were consistently significantly dysregulated in at least two included studies. Three studies found that miR-126 was upregulated in extracellular vesicles but downregulated in total plasma (26, 33, 50), which is consistent with Park’s systematic review (2018). This finding could be explained by the loss of kidney reabsorption and subsequent excretion, leading to the depletion of circulating miR-126 (19). miR-216 participates in maintaining endothelial cells and vascular hemostasis. It enhances vascular endothelial growth factor (VEGF) signaling (53, 54). The dysregulation of miR-126 indicates that it might be related to the progression of diabetes and altered during kidney damage (32, 33). Two studies found the upregulation of miR-145, the glomerular marker of mesangial cells (33). However, the role of this miR-145 in renal tissue damage remains unclear (33). miR-150 promotes renal fibrosis, and it was upregulated in both serum and urine samples in DKD patients compared to controls (36, 48). miR-21-5p was upregulated in urinary exosomes in DKD patients and correlated with creatinine and eGFR (49, 52), which is consistent with the review by Gholaminejad et al. (9). This mRNA participates in activating transforming growth factor (TGF)-β, which works in glomerular cell proliferation and matrix expansion, contributing to renal failure (55). Five studies analyzed the diagnostic value of miRNA (28, 42, 47, 49, 51). Compared to single miRNA biomarkers (i.e., miR-192, miR-194, miR-215, miR-615-3p, miR-3147, miR-4534), a combination of miRNAs (i.e., let-7i-5p, miR-15b-5p, miR-24-3p, and miR-27b-3p) or miRNA in combination with other clinical characteristics (i.e., miR-615-3p and ACR; miR-30b-5p, miR-21-5p, age, gender, and HDL-C) seemed to have higher accuracy in predicting DKD. Our review identified different miRNAs in DKD studies; future studies should confirm the most accurate and stable miRNA biomarker for DKD, and the diagnostic value of miRNA in combination with other clinical indicators should also be further explored.

For mRNA, two upregulated and four downregulated mRNAs were identified in the included studies, and the upregulation of WT1 in DKD was demonstrated in two studies. WT1 is the transcriptional regulator of genes related to growth and apoptosis and is vital in embryogenesis during kidney development (56). Regarding diagnostic value, WT1 seems to have a higher accuracy in predicting overt DKD (AUC: 0.705–0.83) than incipient DKD (AUC: 0.63). Feng et al. found that CCL21 mRNA was an efficient inflammatory marker to differentiate DKD patients without eGFR reduction from non-DKD diabetic patients. Also, its predictive ability was better than standard indicators (i.e., ACR and eGFR). Furthermore, CCL21 mRNA (AUC: 0.888–1.0) seemed to have better accuracy than other mRNAs (i.e., CDH2, MCP-1, and ACE; AUC: 0.61–0.75). Our review identified seven dysregulated proteins, and the diagnostic value was investigated in four of them. The WT1 protein in urine exosomes can effectively predict an early reduction in GRF (AUC: 0.92) (29). As previously mentioned, WT1 has been associated with podocyte malfunction and can be used as a marker for podocyte damage (57). Additionally, the WT1 protein seemed more accurate and sensitive in diagnosing DKD than WT1 mRNA; future studies with head-to-head comparisons are needed to confirm this finding. CALM1 is a regulatory protein for cell motility, differentiation, and proliferation. It was also found to have an excellent diagnostic value for DKD in combination with serum albumin levels (38, 58). The same research team identified the upregulated PAK6 and EGFR as diagnostic biomarkers of DKD (39). While the relationship between PAK6 and DKD is not well understood, the role of EGFR in the pathogenesis of DKD has been extensively studied. Elevated glucose levels activate EGFR and contribute to multicellular dysfunction, which triggers and accelerates kidney injury (59, 60). EGFR in combination with PAK6 has good predictive value and sensitivity (AUC: 0.897) (39).

An increasing number of studies have revealed a significant interest in exosomes for diagnosing and treating DKD, which presents both opportunities and challenges. First, the development of exact and non-invasive diagnostic methods is still of great importance. Due to the complexity of sources and cargoes, one obstacle to applying exosomes in DKD diagnosis is the discrepancy between the sensitivity and specificity of cargoes in diagnosing various kidney-related diseases. Finding reliable and specific exosomal RNAs and/or proteins may be beneficial for the widespread application of exosomes in diagnosing DKD, especially for urinary exosomes. In general, plasma exosomes may not pass through the glomerular filtration barrier. Moreover, the exosomes are protected by their bilayer membrane structure. Thus, urinary exosomes reflect the physiopathological state of the kidney other than the serum or circulation (61). Second, exosomes involving “long-distance” intercellular communication underlying pathogenesis may provide some novel clues to reveal the pathological mechanisms of DKD.

The present study has some limitations. First, the sample sizes of cases and controls were not always matched, and there were some baseline differences between groups, including age and blood pressure. Although hypertension is associated with DKD, it could be a confounding factor that was seldom adjusted in the included studies (62). Second, the heterogeneity of the included studies was high as they reported many different exosomes using diverse samples and methods. Future meta-analysis studies with more homogeneous studies (i.e., the outcome of the same exosome) are needed to confirm the reliability of the diagnostic results. Third, not all the included studies reported the sensitivity and specificity of the diagnostic test. Nonetheless, this review demonstrated that exosomes, especially in combination with other exosomes or clinical indicators, may be suitable as diagnostic biomarkers of DKD. More clinical data are required in the future to verify this finding.

## Conclusion

This is the first study that reviewed the role of exosomes in the pathogenesis and management of DKD and the first meta-analysis on the diagnostic values of exosomes in DKD. The included exosomes had an AUC of 0.70 (95% CI: 0.59–0.80), sensitivity of 0.79 (95% CI: 0.70–0.85), and specificity of 0.82 (95% CI: 0.78–0.85), indicating that exosomes, as a non-invasive method, may be appropriate for use as diagnostic biomarkers of DKD. Moreover, panels of exosomes or the combination of exosomes with other clinical indicators seemed more accurate than single exosomes.

## Data Availability

The original contributions presented in the study are included in the article/**Supplementary Material**. Further inquiries can be directed to the corresponding author.
