# Supplementary material for: The role of exosomes in the pathogenesis and management of diabetic kidney disease: a systematic review and meta-analysis
Source: Front Endocrinol (Lausanne). 2024 Dec 3;15:1398382. doi: 10.3389/fendo.2024.1398382 (PMC11658263; doi:10.3389/fendo.2024.1398382)
Supplement: Supplementary file 1 [file Table1.docx]

**Supplementary appendix 1. Search strategies**

**Search strategy of PubMed**

| NO. | Search Details | Results |
| --- | --- | --- |
| #6 | (#1 OR #2) AND (#3 OR #4) Filters: Humans | 427 |
| #5 | (#1 OR #2) AND (#3 OR #4) | 727 |
| #4 | (((((((((((((((((Diabetic Nephropathy) OR (Diabetic Kidney Disease)) OR (Diabetic Kidney Diseases)) OR (Diabetic Glomerulosclerosis)) OR (Intracapillary Glomerulosclerosis)) OR (Nodular Glomerulosclerosis)) OR (Kimmelstiel-Wilson Syndrome)) OR (Kimmelstiel Wilson Syndrome)) OR (Kimmelstiel-Wilson Disease)) OR (Kimmelstiel Wilson Disease)) OR (diabetes nephropathy)) OR (diabetic glomerulopathy)) OR (diabetic intercapillary glomerulosclerosis)) OR (diabetic nephropathies)) OR (diabetic nephrosclerosis)) OR (intercapillary glomerulosclerosis)) OR (Kimmelstiehl Wilson syndrome)) OR (kimmelstiel wilson nephropathy) | 107,492 |
| #3 | "Diabetic Nephropathies"[Mesh] | 29,847 |
| #2 | ((((((((((((((((((((((((((((((((((((((((((((exosomes) OR (extracellular vesicle)) OR (extracellular vesicles)) OR (exosome)) OR (cell derived microparticle)) OR (cell derived microparticles)) OR (cell-derived microparticles)) OR (circulating microparticle)) OR (circulating microparticles)) OR (membrane microparticles)) OR (microvesicle)) OR (procoagulant microparticle)) OR (procoagulant microparticles)) OR (membrane microparticle)) OR (Endosome)) OR (Receptosomes)) OR (Receptosome)) OR (Cell-Derived Microparticle)) OR (Ectosomes)) OR (Ectosome)) OR (Shedding Microvesicles)) OR (Shedding Microvesicle)) OR (Cell Membrane Microparticles)) OR (Cell Membrane Microparticle)) OR (Circulating Cell-Derived Microparticles)) OR (Circulating Cell Derived Microparticles)) OR (Circulating Cell-Derived Microparticle)) OR (RNA Exosome)) OR (RNA Exosome Complex)) OR (Cytoplasmic Exosome Complex)) OR (Secretory Vesicle)) OR (Secretory Granules)) OR (Secretory Granule)) OR (Synaptic-Like Microvesicles)) OR (Synaptic-Like Microvesicle)) OR (Synaptic Like Microvesicles)) OR (Synaptic Like Microvesicle)) OR (SLMVs)) OR (Condensing Vacuoles)) OR (Condensing Vacuole)) OR (Zymogen Granules)) OR (Zymogen Granule)) OR (exosome multienzyme ribonuclease complex)) OR (secretion vesicle)) OR (secretory vesicles) | 192,782 |
| #1 | (((("Exosomes"[Mesh]) OR "Endosomes"[Mesh]) OR "Cell-Derived Microparticles"[Mesh]) OR "Exosome Multienzyme Ribonuclease Complex"[Mesh]) OR "Secretory Vesicles"[Mesh] | 43,199 |

**Search strategy of EMBASE**

| No. | Query | Results |
| --- | --- | --- |
| #8 | (#1 OR #2 OR #3 OR #4 OR #5) AND (#6 OR #7) | 563 |
| #7 | 'diabetic nephropathy':ti,ab,kw OR 'diabetic kidney disease':ti,ab,kw OR 'diabetic kidney diseases':ti,ab,kw OR 'diabetic glomerulosclerosis':ti,ab,kw OR 'intracapillary glomerulosclerosis':ti,ab,kw OR 'nodular glomerulosclerosis':ti,ab,kw OR 'kimmelstiel-wilson syndrome':ti,ab,kw OR 'kimmelstiel wilson syndrome':ti,ab,kw OR 'kimmelstiel-wilson disease':ti,ab,kw OR 'kimmelstiel wilson disease':ti,ab,kw OR 'diabetes nephropathy':ti,ab,kw OR 'diabetic glomerulopathy':ti,ab,kw OR 'diabetic intercapillary glomerulosclerosis':ti,ab,kw OR 'diabetic nephropathies':ti,ab,kw OR 'diabetic nephrosclerosis':ti,ab,kw OR 'intercapillary glomerulosclerosis':ti,ab,kw OR 'kimmelstiehl wilson syndrome':ti,ab,kw OR 'kimmelstiel wilson nephropathy':ti,ab,kw | 42660 |
| #6 | 'diabetic nephropathy'/exp | 56892 |
| #5 | 'exosomes':ti,ab,kw OR 'extracellular vesicle':ti,ab,kw OR 'extracellular vesicles':ti,ab,kw OR 'exosome':ti,ab,kw OR 'cell derived microparticle':ti,ab,kw OR 'cell derived microparticles':ti,ab,kw OR 'cell-derived microparticles':ti,ab,kw OR 'circulating microparticle':ti,ab,kw OR 'circulating microparticles':ti,ab,kw OR 'membrane microparticles':ti,ab,kw OR 'microvesicle':ti,ab,kw OR 'procoagulant microparticle':ti,ab,kw OR 'procoagulant microparticles':ti,ab,kw OR 'membrane microparticle':ti,ab,kw OR 'endosome':ti,ab,kw OR 'receptosomes':ti,ab,kw OR 'receptosome':ti,ab,kw OR 'cell-derived microparticle':ti,ab,kw OR 'ectosomes':ti,ab,kw OR 'ectosome':ti,ab,kw OR 'shedding microvesicles':ti,ab,kw OR 'shedding microvesicle':ti,ab,kw OR 'cell membrane microparticles':ti,ab,kw OR 'cell membrane microparticle':ti,ab,kw OR 'circulating cell-derived microparticles':ti,ab,kw OR 'circulating cell derived microparticles':ti,ab,kw OR 'circulating cell-derived microparticle':ti,ab,kw OR 'rna exosome':ti,ab,kw OR 'rna exosome complex':ti,ab,kw OR 'cytoplasmic exosome complex':ti,ab,kw OR 'secretory vesicle':ti,ab,kw OR 'secretory granules':ti,ab,kw OR 'secretory granule':ti,ab,kw OR 'synaptic-like microvesicles':ti,ab,kw OR 'synaptic-like microvesicle':ti,ab,kw OR 'synaptic like microvesicles':ti,ab,kw OR 'synaptic like microvesicle':ti,ab,kw OR 'slmvs':ti,ab,kw OR 'condensing vacuoles':ti,ab,kw OR 'condensing vacuole':ti,ab,kw OR 'zymogen granules':ti,ab,kw OR 'zymogen granule':ti,ab,kw OR 'exosome multienzyme ribonuclease complex':ti,ab,kw OR 'secretion vesicle':ti,ab,kw OR 'secretory vesicles':ti,ab,kw | 86339 |
| #4 | 'secretory vesicle'/exp | 3053 |
| #3 | 'exosome multienzyme ribonuclease complex'/exp | 177 |
| #2 | 'membrane microparticle'/exp | 10349 |
| #1 | 'exosome'/exp | 58620 |

**Search strategy of Cochrane Library**

| NO. | Search deatiles | Hits |
| --- | --- | --- |
| #1 | MeSH descriptor: [Exosomes] explode all trees | 28 |
| #2 | MeSH descriptor: [Endosomes] explode all trees | 6 |
| #3 | MeSH descriptor: [Cell-Derived Microparticles] explode all trees | 86 |
| #4 | MeSH descriptor: [Exosome Multienzyme Ribonuclease Complex] explode all trees | 1 |
| #5 | MeSH descriptor: [Secretory Vesicles] explode all trees | 6 |
| #6 | (exosomes):ti,ab,kw OR (extracellular vesicle):ti,ab,kw OR (extracellular vesicles):ti,ab,kw OR (exosome):ti,ab,kw OR (cell derived microparticle):ti,ab,kw OR (cell derived microparticles):ti,ab,kw OR (cell-derived microparticles):ti,ab,kw OR (circulating microparticle):ti,ab,kw OR (circulating microparticles):ti,ab,kw OR (membrane microparticles):ti,ab,kw OR (microvesicle):ti,ab,kw OR (procoagulant microparticle):ti,ab,kw OR (procoagulant microparticles):ti,ab,kw OR (membrane microparticle):ti,ab,kw OR (Endosome):ti,ab,kw OR (Receptosomes):ti,ab,kw OR (Receptosome):ti,ab,kw OR (Cell-Derived Microparticle):ti,ab,kw OR (Ectosomes):ti,ab,kw OR (Ectosome):ti,ab,kw OR (Shedding Microvesicles):ti,ab,kw OR (Shedding Microvesicle):ti,ab,kw OR (Cell Membrane Microparticles):ti,ab,kw OR (Cell Membrane Microparticle):ti,ab,kw OR (Circulating Cell-Derived Microparticles):ti,ab,kw OR (Circulating Cell Derived Microparticles):ti,ab,kw OR (Circulating Cell-Derived Microparticle):ti,ab,kw OR (RNA Exosome):ti,ab,kw OR (RNA Exosome Complex):ti,ab,kw OR (Cytoplasmic Exosome Complex):ti,ab,kw OR (Secretory Vesicle):ti,ab,kw OR (Secretory Granules):ti,ab,kw OR (Secretory Granule):ti,ab,kw OR (Synaptic-Like Microvesicles):ti,ab,kw OR (Synaptic-Like Microvesicle):ti,ab,kw OR (Synaptic Like Microvesicles):ti,ab,kw OR (Synaptic Like Microvesicle):ti,ab,kw OR (SLMVs):ti,ab,kw OR (Condensing Vacuoles):ti,ab,kw OR (Condensing Vacuole):ti,ab,kw OR (Zymogen Granules):ti,ab,kw OR (Zymogen Granule):ti,ab,kw OR (exosome multienzyme ribonuclease complex):ti,ab,kw OR (secretion vesicle):ti,ab,kw OR (secretory vesicles):ti,ab,kw | 818 |
| #7 | MeSH descriptor: [Diabetic Nephropathies] explode all trees | 1807 |
| #8 | (Diabetic Nephropathy):ti,ab,kw OR (Diabetic Kidney Disease):ti,ab,kw OR (Diabetic Kidney Diseases):ti,ab,kw OR (Diabetic Glomerulosclerosis):ti,ab,kw OR (Intracapillary Glomerulosclerosis):ti,ab,kw OR (Nodular Glomerulosclerosis):ti,ab,kw OR (Kimmelstiel-Wilson Syndrome):ti,ab,kw OR (Kimmelstiel Wilson Syndrome):ti,ab,kw OR (Kimmelstiel-Wilson Disease):ti,ab,kw OR (Kimmelstiel Wilson Disease):ti,ab,kw OR (diabetes nephropathy):ti,ab,kw OR (diabetic glomerulopathy):ti,ab,kw OR (diabetic intercapillary glomerulosclerosis):ti,ab,kw OR (diabetic nephropathies):ti,ab,kw OR (diabetic nephrosclerosis):ti,ab,kw OR (intercapillary glomerulosclerosis):ti,ab,kw OR (Kimmelstiehl Wilson syndrome):ti,ab,kw OR (kimmelstiel wilson nephropathy):ti,ab,kw | 7405 |
| #9 | (#1 OR #2 OR #3 OR #4 OR #5 OR #6) AND (#7 OR #8) | 13 |

**Search strategy of web of science**

| NO. | Search deatiles | Hits |
| --- | --- | --- |
| #1 | (((((((((((((((((((((((((((((((((((((((((((TS=(exosomes) OR TS=(extracellular vesicle)) OR TS=(extracellular vesicles)) OR TS=(exosome)) OR TS=(cell derived microparticle)) OR TS=(cell derived microparticles)) OR TS=(cell-derived microparticles)) OR TS=(circulating microparticle)) OR TS=(circulating microparticles)) OR TS=(membrane microparticles)) OR TS=(microvesicle)) OR TS=(procoagulant microparticle)) OR TS=(procoagulant microparticles)) OR TS=(membrane microparticle)) OR TS=(Endosome)) OR TS=(Receptosomes)) OR TS=(Receptosome)) OR TS=(Cell-Derived Microparticle)) OR TS=(Ectosomes)) OR TS=(Ectosome)) OR TS=(Shedding Microvesicles)) OR TS=(Shedding Microvesicle)) OR TS=(Cell Membrane Microparticles)) OR TS=(Cell Membrane Microparticle)) OR TS=(Circulating Cell-Derived Microparticles)) OR TS=(Circulating Cell Derived Microparticles)) OR TS=(Circulating Cell-Derived Microparticle)) OR TS=(RNA Exosome)) OR TS=(RNA Exosome Complex)) OR TS=(Cytoplasmic Exosome Complex)) OR TS=(Secretory Vesicle)) OR TS=(Secretory Granules)) OR TS=(Secretory Granule)) OR TS=(Synaptic-Like Microvesicles)) OR TS=(Synaptic-Like Microvesicle)) OR TS=(Synaptic Like Microvesicles)) OR TS=(Synaptic Like Microvesicle)) OR TS=(SLMVs)) OR TS=(Condensing Vacuoles)) OR TS=(Condensing Vacuole)) OR TS=(Zymogen Granules)) OR TS=(Zymogen Granule)) OR TS=(exosome multienzyme ribonuclease complex)) OR TS=(secretion vesicle)) OR TS=(secretory vesicles) | 103340 |
| #2 | ((((((((((((((((TS=(Diabetic Nephropathy) OR TS=(Diabetic Kidney Disease)) OR TS=(Diabetic Kidney Diseases)) OR TS=(Diabetic Glomerulosclerosis)) OR TS=(Intracapillary Glomerulosclerosis)) OR TS=(Nodular Glomerulosclerosis)) OR TS=(Kimmelstiel-Wilson Syndrome)) OR TS=(Kimmelstiel Wilson Syndrome)) OR TS=(Kimmelstiel-Wilson Disease)) OR TS=(Kimmelstiel Wilson Disease)) OR TS=(diabetes nephropathy)) OR TS=(diabetic glomerulopathy)) OR TS=(diabetic intercapillary glomerulosclerosis)) OR TS=(diabetic nephropathies)) OR TS=(diabetic nephrosclerosis)) OR TS=(intercapillary glomerulosclerosis)) OR TS=(Kimmelstiehl Wilson syndrome)) OR TS=(kimmelstiel wilson nephropathy) | 54362 |
| #3 | #2 AND #1 | 409 |
